# Retinal microvascular features and cognitive change in the Lothian-Birth Cohort 1936

**DOI:** 10.1016/j.dadm.2019.04.012

**Published:** 2019-07-10

**Authors:** Sarah McGrory, Lucia Ballerini, Judith A. Okely, Stuart J. Ritchie, Fergus N. Doubal, Alex S.F. Doney, Baljean Dhillon, John M. Starr, Thomas J. MacGillivray, Emanuele Trucco, Joanna M. Wardlaw, Ian J. Deary

**Affiliations:** a VAMPIRE project, Center for Clinical Brain Sciences, Edinburgh Medical School, University of Edinburgh, Edinburgh, UK; b Department of Psychology, University of Edinburgh, Edinburgh, UK; c Center for Cognitive Ageing and Cognitive Epidemiology, Department of Psychology, University of Edinburgh, Edinburgh, UK; d Social, Genetic and Developmental Psychiatry Centre, Institute of Psychiatry, Psychology and Neuroscience, King's College London, London, UK; e Division of Cardiovascular and Diabetes Medicine, Medical Research Institute, Ninewells Hospital and Medical School, Dundee, UK; f Alzheimer Scotland Dementia Research Centre, Department of Psychology, University of Edinburgh, Edinburgh, UK; g VAMPIRE Project, Computing, School of Science and Engineering, University of Dundee, Dundee, UK; h Scottish Imaging Network, A Platform for Scientific Excellence (SINAPSE) Collaboration, Edinburgh, UK; i UK Dementia Research Institute at the University of Edinburgh, Edinburgh, UK

**Keywords:** Retinal imaging, Cognitive change, Longitudinal study

## Abstract

**Introduction:**

We test whether measures of the retinal vasculature are associated with cognitive functioning and cognitive change.

**Methods:**

Retinal images from a narrow-age cohort were analyzed using Vessel Assessment and Measurement Platform for Images of the Retina, producing a comprehensive range of quantitative measurements of the retinal vasculature, at mean age 72.5 years (SD = 0.7). Cognitive ability and change were measured using a battery of multiple measures of memory, visuospatial, processing speed, and crystallized cognitive abilities at mean ages 73, 76, and 79 years. We applied multivariate growth curve models to test the association between retinal vascular measurements with cognitive abilities and their changes.

**Results:**

Almost all associations were nonsignificant. In our most parsimonious model, venular asymmetry factor was associated with speed at age 73.

**Discussion:**

Our null findings suggest that the quantitative retinal parameters applied in this study are not significantly associated with cognitive functioning or cognitive change.

## 1 Introduction

Owing to the homology between retinal and brain microvasculature, it has been hypothesized that retinal vessels might act as a surrogate marker of the brain microvasculature, with age-related changes to the retinal vessels providing an indirect marker of homologous changes to the vessels within the brain [1]. It has also been proposed that age-related retinal microvasculature changes might be associated with cognitive decline, providing an approximation of the impact on cerebral small-vessel disease on cognitive functioning [2]. With the growing interest in the application of retinal vasculature to gain insight into the state of systemic and brain microcirculation and, perhaps, thereby to later-life cognitive functioning, it is critical to determine which, if any, retinal measurements are associated with cognitive functioning and cognitive change in later life.

Cross-sectional studies examining features of retinal microvascular pathological changes and cognitive outcomes (delayed word recall and digit symbol substitution scores) have reported generally consistent, modest associations (odds ratio [OR]: 2.6; 95% confidence interval [CI] = 1.3–2.91; OR: 2.18; 95% CI = 1.02–4.64) [2], [3]. Evidence of associations between quantitative nonpathological retinal microvascular measurements and cognitive aging has been mixed. Lower fractal dimension of the retinal vasculature was associated with cognitive dysfunction (OR: 1.71; 95% CI = 1.03-2.82) in the Singapore Malay Eye Study [4]. The Blue Mountains Eye Study reported an association between venular dilation and cognitive impairment (OR: 1.8; 95% CI = 1.0-3.2) [5]. However, other studies have found little evidence that such quantitative retinal microvascular measurements are associated with nonpathological cognitive aging [6], [7], [8], [9].

Longitudinal studies examining retinal microvasculature changes in relation to cognitive changes have found some evidence for the role of microvascular disease in cognitive decline, with marginal associations between retinal features and cognitive decline. Retinal microvascular pathology in particular has been associated with an increased risk of cognitive decline. The Atherosclerosis Risk in Communities Study found declines in processing speed and executive functioning and attention (OR: 2.18 and 1.33, respectively) over 14 years [2], with a follow-up study reporting difference in 20-year cognitive change for moderate/severe versus no retinopathy (standard deviation [SD]: −0.53, 95% CI = −0.74 to −0.33) [10]. Haan et al. reported poor modified Mini-Mental State Examination scores (mean difference: 1.01, standard error: 0.43) in those with retinopathy [11]. The few studies examining vessel width in relation to cognitive change have found little evidence to support an association between arteriolar and venular diameters and cognitive decline [2], [10], [12].

To our knowledge, no studies have been published to date examining trajectories of different major cognitive domains and their associations with a wide range of retinal vascular features. Most longitudinal studies have focused on specific retinal measures such as retinopathy signs (microaneurysms, soft or hard exudates, retinal hemorrhages, macular edema, intraretinal microvascular abnormalities, venous beading, new vessels, vitreous hemorrhage, disc swelling, or laser photocoagulation scars) [2], [11], [13], [14], [15] or arteriolar narrowing, focal narrowing, or arteriolar nicking [2], [13], [14]; the present study analyzed a wide range of quantitative retinal vascular parameters including both local and global vascular topographic features (including branching angles of vessels, tortuosity, and fractal dimension). These features are thought to reflect how optimally arranged and developed the retinal microvasculature is and therefore may indicate the state of the cerebral microcirculation [16]. These changes are milder than pathological signs of retinopathy but are more common, which increases the prognostic value of these features should an association be found. We assessed cognitive functioning levels at age 73 and cognitive changes from age 73 to age 79 in a range of highly sensitive cognitive tests measuring four distinct domains of cognition using multiple tests for each domain: memory, processing speed, visuospatial ability, and crystallized ability [17]. The present study, therefore, aims to test which, if any, retinal vascular measurements are associated with between-person variation in cognitive levels at baseline and predict future changes, both in specific, major cognitive ability domains and more generally across all cognitive abilities, between age ∼73 to 79 years, after controlling for a range of relevant covariates.

## 2 Methods

### 2.1 Participants

The Lothian Birth Cohort 1936 (LBC1936) is a prospective study of a narrow-age sample of community-dwelling individuals, living mostly in the city of Edinburgh and the surrounding Lothian area of Scotland, UK [18], [19], [20]. All participants were born in 1936, and most took a general mental ability test in the Scottish mental survey of 1947 at age 11. Those living in the Edinburgh area at about 70 years of age were invited to be recruited in the LBC1936 longitudinal study. Participants were followed up in 2004-2007 (wave 1: mean age: 69.5 years, SD = .08; n = 1,091, 543 women), again in 2007-2011 (wave 2: mean age: 72.5 years, SD = 0.07; n = 866, 418 women), again in 2011-2013 (wave 3: mean age: 76.3 years, SD = 0.07; n = 697, 337 women), and again in 2014-2017 (wave 4: mean age: 79.3 years, SD = 0.6; n = 550, 275 women). Retinal imaging was performed at wave 2 at a mean age of about 73 years; cognitive, physical, and health assessments were performed concurrently and again at waves 3 and 4, at mean ages of 76 and 79 years, respectively. Those with retinal measurements from both right and left eyes formed the analytic sample for the present study (n = 603). Retinal imaging and all data analyses were performed blind to all other results including cognitive ability.

Written informed consent was obtained from all participants. The research was carried out in compliance with the Helsinki Declaration. The study was approved by the Multicentre Research Ethics Committee for Scotland (MREC/01/01/56; 07/MRE00/58) and the Lothian Research Ethics Committee (LREC/2003/3/29).

### 2.2 Measures

#### 2.2.1 Retinal measurements

Digital fundus retinal images of the right and left retinas were captured using a nonmydriatic camera and a 45° field of view (CR-DGi; Canon USA, Inc., Lake Success, NY). Images were analyzed by a trained grader (S.M.) at the University of Edinburgh, using the semiautomated software Vessel Assessment and Measurement Platform for Images of the Retina (VAMPIRE) [21], [22], [23]. A total of 814 participants had retinal images of both eyes taken at wave 2. Retinal parameters from these images were measured for each of the 680 participants whose images were of sufficient quality for analysis using VAMPIRE, which resulted in a sample of 603 with retinal measurements of both eyes. Supplementary Fig. S1 shows a flowchart of how the analytic sample for the present study was derived. The main reasons for image rejection included images being centered overly toward the macula resulting in too few visible vessels; images with known pathologies, including cataract and asteroid hyalosis; images being of very poor quality, including out-of-focus images; small pupil size leading to dark images; and overexposure. In brief, retinal vascular parameters were measured from the vessel caliber—central retinal artery equivalent, central retinal vein equivalent, and the variation in caliber—the standard deviation of arteriolar and venular widths; measures of branching complexity—arteriolar and venular fractal dimension; measures of vessel tortuosity—arteriolar and venular tortuosity; and measures of arteriolar and venular branching geometry—branching coefficient, length-diameter ratio, and asymmetry factor. Fourteen measurements were calculated from each retinal image. These measurements were selected from a larger range of VAMPIRE measurements through a data reduction process described in detail previously [24]. To reduce the number of variables, reduce multicollinearity, and increase reliability, the abovementioned measurements from both eyes of each participant were averaged to provide a mean measurement for all variables. A description of all parameters is listed in Supplementary Table S1. Separate arteriolar and venular measures are indicated by lowercase “a” or “v”.

### 2.2.2 Cognitive tests

All cognitive tests and administration procedures have been described in detail previously [18]. Following previous analyses on this battery of cognitive tests [17], the measurement of cognitive ability was grouped into four domains designed to assess multiple aspects of cognitive functioning: memory, processing speed, visuospatial ability, and crystallized intelligence. Memory was measured by logical memory, verbal paired associates, and digit span backwards from the Wechsler Memory Scale Third Edition [25]. Processing speed was measured by symbol search and digit symbol from the Wechsler Adult Intelligence Scale Third Edition (WAIS-III) [26]. Choice Reaction Time and Inspection Time: inspection time and choice reaction time tests and procedures have been described in detail [27], [28]. Visuospatial ability was measured with block design and matrix reasoning from the WAIS-III and spatial span forward and spatial span backward from the WMS-III. Crystallized intelligence was measured by the Wechsler Test of Adult Reading [29], the National Adult Reading Test [30], and phonemic verbal fluency test [31].

### 2.2.3 Covariates

Age (in days at time of retinal imaging and cognitive testing) and sex were included as covariates. Heath assessments carried out at testing included self-reported medical history (recording histories [yes, no] of hypertension, hypercholesterolemia, and diabetes), and smoking status, (current vs. ex or never). Systolic and diastolic blood pressure (average of three sitting measures), plasma hemoglobin A1c, and total serum cholesterol were also measured during physical assessment with a research nurse. Including these covariates, which have been previously linked to cognitive aging and retinal measurements, allowed us to determine if they have any confounding effects on the hypothesized association between retinal measurements and cognitive ability.

### 2.6 Statistical analysis

Data were analyzed using a multivariate latent growth curve approach, implemented in Mplus (version 7.4) [32] using full-information maximum likelihood estimation to take all data into account. The model estimated the overall level of each cognitive test (the intercept at mean age 73 years) and the slope of its change across the three measurement waves (the trajectory between age 73 and 79 years). In doing so the model estimated the association of each retinal vascular feature with the overall level of each cognitive test and the slope of its change. The average time lag between the waves (3.77 years from waves 2 to 3, and 6.87 years from waves 2 to 4) was used as the path weights for calculation of the slope factor, with the path from the slope factor to the initial wave's (wave 2) test score being set to zero. A ‘factor of curves’ model was used with latent level and slope factors analyzed as if they were directly measured variables, allowing their organization into higher-order factor structures to be investigated and their relations with covariates [33].

A single general factor was estimated from the growth curve levels for each test. We then tested whether it was possible to extract a general factor of cognitive change from the growth curve slopes. Model fit was tested using four indices of absolute fit: root mean square error of approximation (values < 0.06 considered acceptable), comparative fit index (values > 0.95 considered acceptable), Tucker-Lewis index (values > 0.95 considered acceptable), and standardized root mean square residual (values < 0.08 considered acceptable).

We ran two variants of this model: first, we adjusted for age and sex; and second, we additionally adjusted for vascular risk factors (VRF). Vascular risk was measured by systolic and diastolic blood pressure, blood cholesterol, blood hemoglobin A1c, hypertension, hypercholesterolemia, and smoking. This allowed us to compare the association between retinal measurements and cognition with and without controlling for VRF.

The large number of significance tests in these models increases the potential for type I errors. For that reason, *P* values were corrected for multiple comparisons using Hochberg's false discovery rate (FDR) correction [34].

## 3 Results

There were 603 participants of mean age 72.5 years (SD = 0.7) at wave 2, 492 participants of mean age 76.2 years (SD = 0.7) at wave 3, and 391 participants of mean age 79.4 years (SD = 0.7) at wave 4. Table 1 provides the cognitive test results and vascular risk factor findings at each wave.

**Table 1:** Descriptive statistics for each cognitive test and covariate at each testing wave

Variable	Age 73 (wave 2)	Age 76 (wave 3)	Age 79 (wave 4)
N	603	492	391
Sex			
Female	300 (49.8)	242 (49.2)	199 (50.9)
Male	303 (50.2)	250 (50.8)	192 (49.1)
Age	72.5 (0.7)	76.25 (0.67)	79.36 (0.70)

Cognitive tests	n	M	SD	n	M	SD	n	M	SD
Visuospatial									
Matrix reasoning	603	13.23	5.00	487	13.04	4.94	383	12.98	5.02
Block design	602	33.89	10.07	489	32.30	9.97	382	31.19	9.86
Spatial span	601	7.36	1.39	488	7.33	1.40	385	7.10	1.39
Crystallized									
NART	603	34.50	8.29	492	35.24	7.92	389	35.83	8.15
WTAR	603	41.22	6.95	492	41.30	6.91	389	41.84	6.97
Verbal fluency	603	43.61	12.94	491	43.36	12.75	389	43.79	13.42
Verbal paired associates	601	74.08	17.92	484	74.05	19.51	385	72.71	19.80
Memory									
Logical memory	589	27.28	9.32	469	26.24	9.75	358	27.07	9.46
Digit span backward	603	7.85	2.29	490	7.80	2.36	389	7.64	2.16
Symbol search	603	24.91	6.00	488	24.83	6.48	381	22.80	6.70
Speed									
Digit symbol	602	57.20	11.93	488	54.27	12.74	385	51.58	12.85
Inspection time	591	111.77	11.64	466	110.64	12.57	337	106.76	14.06
Choice reaction time	603	0.64	0.08	487	0.68	0.10	388	0.70	0.11
Covariates									
HbA1C	575	5.72	0.60	349	5.84	0.63	10	5.69	0.20
Cholesterol	581	5.24	1.16	451	5.04	1.22	371	5.04	1.20
Systolic blood pressure	600	148.48	18.99	489	147.86	19.57	388	145.14	18.99
Diastolic blood pressure	600	78.22	9.59	489	78.88	10.09	388	76.85	9.90
Covariates (dichotomous)	n total	n present	n absent	n total	n present	n absent	n total	n present	n absent

Smoker/ex-smoker	603	303	300	491	234	257	391	179	212
Stroke	603	33	570	492	47	445	389	47	342
Cardiovascular disease	603	161	442	492	163	328	390	139	251
Hypertension	603	270	333	492	256	236	391	218	173
Diabetes	603	58	545	492	58	434	389	51	338
Hypercholesterolemia	603	228	375	489	223	266	388	171	217
APOE e4	574	166	408						

NOTE: The means here are raw and not FIML-estimated. Values are mean (SD) or N (%) unless otherwise stated.

Abbreviations: NART, National Adult Reading Test; WTAR, Wechsler Test of Adult Reading; HbA1C, plasma hemoglobin A1c.

### 3.1 Prediction of study (non)attendance

Table 2 shows differences between participants who provided cognitive ability or retinal imaging data at all three waves (“completers”), n = 391, and those who provided data at only one or two waves (“noncompleters”), n = 212. Those who completed all waves of the study scored higher on all cognitive tests, had lower systolic blood pressure, had lower rates of diabetes, and were less likely to be smokers at age 73 (wave 2). Retinal measurements of completers and noncompleters did not differ, with the exception of arteriolar fractal dimension, where completers had higher fractal dimension, reflecting greater vascular complexity.

**Table 2:** Differences at age 73 between participants who provided data at all three waves (“completers”) and participants who provided data at one or two waves (“noncompleters”)

Characteristics at wave 2	Completers total n = 391	n∗	Noncompleters total n = 212	n†	*P*
Matrix reasoning, M (SD)	13.88 (4.92)	390	12.04 (4.94)	212	<.001
Block design, M (SD)	34.91 (10.01)	390	32.02 (9.93)	212	.001
Spatial span, M (SD)	7.46 (1.35)	389	7.18 (1.43)	212	.017
Paired associates, M (SD)	28.30 (8.87)	384	25.37 (9.96)	205	<.001
Logical memory, M (SD)	75.79 (16.75)	391	70.91 (19.56)	210	.001
Digit span, M (SD)	7.99 (2.34)	391	7.59 (2.16)	212	.040
NART, M (SD)	35.24 (8.10)	391	33.15 (8.47)	212	.003
WTAR, M (SD)	41.86 (6.66)	391	40.04 (7.32)	212	.002
Verbal fluency, M (SD)	44.52 (12.89)	391	41.93 (12.89)	212	.019
Digit symbol, M (SD)	58.67 (11.83)	391	54.49 (11.66)	211	<.001
Symbol search, M (SD)	25.65 (5.84)	391	23.54 (6.08)	212	<.001
Reaction time, M (SD)	−6.36 (0.78)	391	−6.61 (0.92)	212	<.001
Inspection time, M (SD)	112.47 (11.58)	389	110.42 (11.65)	202	.042
CRAE, M (SD)	31.25 (2.23)	391	31.06 (2.17)	212	.335
CRVE, M (SD)	42.21 (3.14)	391	41.94 (3.24)	212	.318
BSTDa, M (SD)	2.17 (0.64)	391	2.27 (0.68)	212	.061
BSTDv, M (SD)	3.96 (0.88)	391	3.91 (0.87)	212	.551
FDa, M (SD)	1.59 (0.05)	391	1.58 (0.05)	212	.008
FDv, M (SD)	1.57 (0.04)	391	1.56 (0.05)	212	.053
TORTa, M (SD)	−9.993 (0.95)	391	−9.969 (0.97)	212	.769
TORTv, M (SD)	−9.911 (0.70)	391	−9.923 (0.68)	212	.841
BCa, M (SD)	2.10 (0.24)	357	2.09 (0.34)	179	.743
BCv, M (SD)	2.08 (1.78)	361	1.96 (0.20)	195	.340
AFa, M (SD)	0.92 (0.04)	357	0.92 (0.05)	179	.890
AFv, M (SD)	0.90 (0.05)	361	0.90 (0.05)	195	.097
LDRa, M (SD)	19.71 (5.33)	357	19.19 (5.22)	179	.280
LDRv, M (SD)	19.15 (4.99)	361	18.66 (5.18)	195	.281
Cholesterol mmol/L, M (SD)	5.29 (1.14)	381	5.14 (1.19)	200	.146
HbA1C, M (SD)	5.70 (0.53)	379	5.76 (0.71)	196	.189
Diastolic BP, M (SD)	77.92 (9.44)	389	78.78 (9.86)	211	.291
Systolic BP, M (SD)	147.34 (17.78)	389	150.57 (20.92)	211	.047
Hypertension, N (%)	165 (42.2)	391	105 (49.5)	212	.084
Diabetes, N (%)	29 (7.4)	391	29 (13.7)	212	.013
High cholesterol, N (%)	149 (38.1)	391	79 (37.3)	212	.838
Smoker/ex-smoker, N (%)	180 (46)	391	123 (58)	212	.005

Abbreviations: NART, National Adult Reading Test; WTAR, Wechsler Test of Adult Reading; CRAE, central retinal arteriolar equivalent; CRVE, central retinal venular equivalent; BSTDa, standard deviation of arteriolar widths in zone B; BSTDv, standard deviation of venular widths in zone B; FDa, arteriolar fractal dimension; FDv, venular fractal dimension; TORTa, arteriolar tortuosity; TORTv, venular tortuosity; BCa, arteriolar branching coefficient; BCv, venular branching coefficient; AFa, arteriolar asymmetry factor; AFv, venular asymmetry factor; LDRa, arteriolar length-to-diameter ratio; LDRv, venular length-to-diameter ratio.

∗ Number of completers with available data at age 73.

† Number of noncompleters with available data at age 73.

### 3.2 Structure of cognitive change

**Fig. 1:**
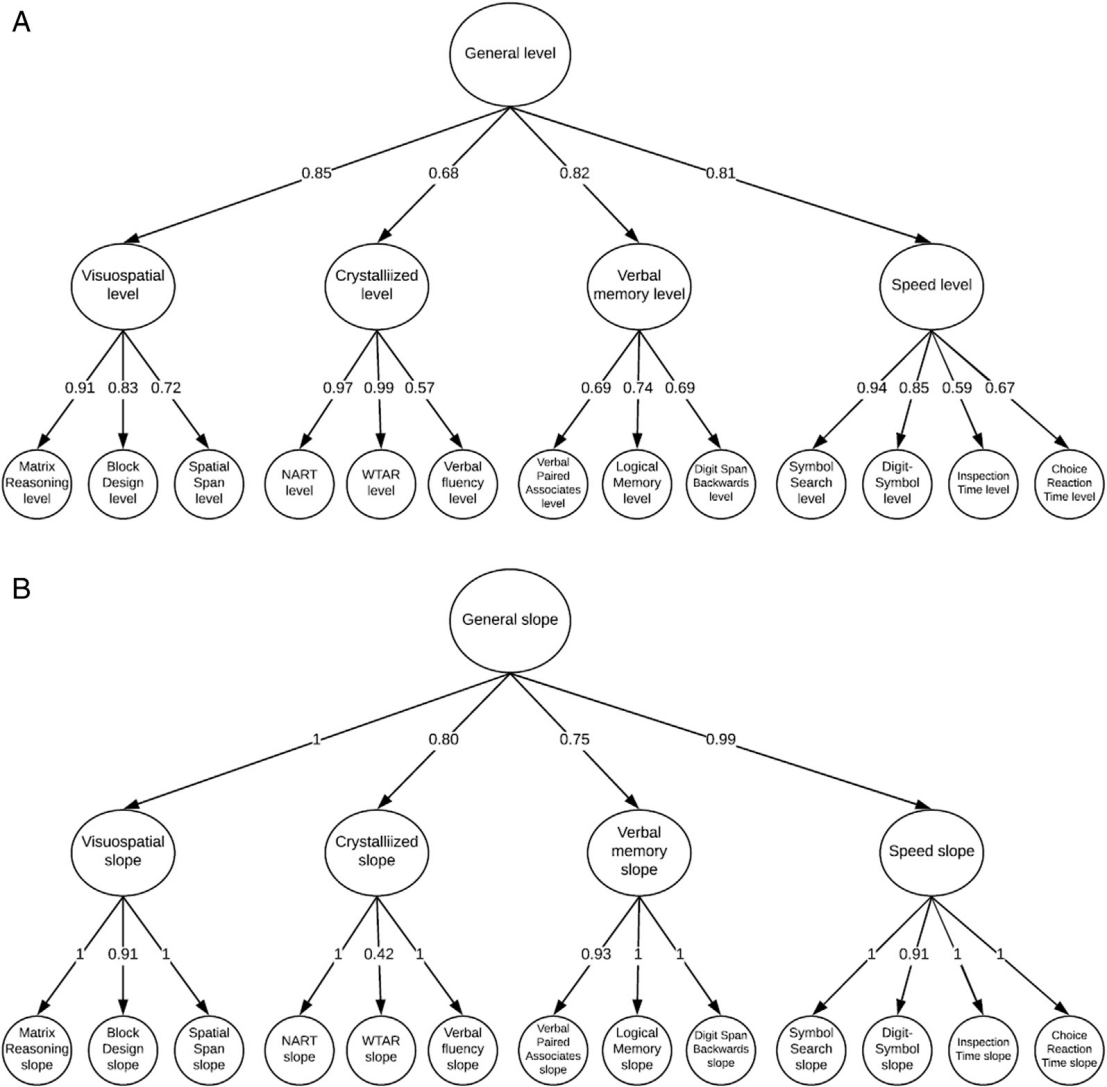
Structural model of cognitive ability levels (A) and slopes (B). The latent levels and slopes of each test are grouped into domains; these domains are grouped under the general factor of cognitive ability. Values are standardized factor loadings. Both A and B were estimated simultaneously in the model.

In our model, nine of the 13 cognitive tests' slopes had specific variances (between person differences in slope) that were near-zero and were estimated as negative, indicating that all variance in change on that test was shared with the aforementioned domain (i.e., they have a standardized loading of 1.0). In addition, the slope of one of the domains (visuospatial) also had negative variance. We fixed the specific variance of these slopes (matrix reasoning, spatial span, National Adult Reading Test, verbal fluency, logical memory, digit span backwards, symbol search, inspection time, choice reaction time, visuospatial) to zero, to allow our model to converge on within-bound estimates (with no negative variance). The model fit well by root mean square error of approximation (0.038), comparative fit index (0.961), Tucker-Lewis index (0.959), and standardized root mean square residual (0.066) (Fig. 1).

### 3.3 Predictors of cognitive level and change

At mean age 73, those with better cognitive function were younger when tested (standardized *β = .*165, *P* < .001). Women tended to perform better than men in tests of crystallized ability (*β* = .217, *P* < .001) and verbal memory (*β* = .459, *P* < .001), and men scored better than women in tests of visuospatial ability (*β* = −.621, *P* < .001).

### 3.4 Retinal predictors of cognitive level and change

Importantly, a large number of the associations were not significant even before correction for multiple comparisons. Across both models, only 18 of the 280 retinal-cognitive function associations were nominally significant. Of these, only one association in the age- and sex-adjusted model survived correction for multiple comparisons; venular asymmetry factor was associated with speed at age 73 (*β* = −.129, *P* < .001, FDR corrected *P* < .001), such that participants with slower processing speed at age 73 tended to have higher asymmetry factor measurements, indicating greater symmetrical widths of daughter branching venules (see Table 3). Eight nominally significant associations in the age- and sex-adjusted model were lost after FDR adjustment (see Table 3). In the age-, sex-, and VRF-adjusted model, none of the 10 nominally significant associations survived FDR correction (all results are shown in Supplementary Table S2 in Supplemental materials).

**Table 3:** Associations of each predictor, entered individually alongside age, sex, with the cognitive level, and slope (cognitive aging from 73 to 79) of cognitive ability from mean age. General factor model and domain models were run separately

Covariate	General factor estimate (SE)		Domain factor estimate (SE)							
	*g* level	*g* slope	Visuospatial level	Crystallized level	Verbal memory level	Speed level	Visuospatial slope	Crystallized slope	Verbal memory slope	Speed slope
Age (baseline)	−**.165 (.044)**∗	−.034 (.054)	.013 (.037)	−.072 (.034)†	.000 (.041)	−.082 (.035)†	.149 (.093)	−.098 (.083)	−.031 (.050)	.011 (.049)
Sex (female)‡	.076 (.097)	.179 (.109)	−**.623 (.064)**∗	**.271 (.068)**∗	**.459 (.079)**∗	.109 (.075)	.136 (191)	.035 (.159)	.140 (.103)	.017 (.102)
CRAE	−.009 (.046)	.082 (.055)	−.018 (.034)	−.033 (.034)	−.026 (.040)	.049 (.035)	.043 (.094)	.014 (.081)	−.028 (.051)	.060 (.050)
CRVE	.065 (.046)	.055 (.055)	−.026 (.033)	.040 (.033)	−.025 (.039)	.041 (.035)	.040 (.092)	−.161 (.084)	.042 (.052)	.044 (.050)
BSTDa	−.019 (.045)	−.021 (.054)	.026 (.034)	−.029 (.034)	.038 (.039)	−.044 (.035)	.052 (.095)	−.051 (.082)	−.057 (.050)	.011 (.049)
BSTDv	.008 (.045)	.042 (.055)	.016 (.033)	−.003 (.034)	−.014 (.040)	.007 (.035)	−.153 (.089)	−.030 (.084)	.020 (.051)	.072 (.050)
FDa	.056 (.047)	.131 (.057)†	−.022 (.035)	−.033 (.035)	−.029 (.041)	.078 (.036)†	.126 (.100)	.093 (.089)	−.035 (.056)	.056 (.054)
FDv	.009 (.045)	.066 (.055)	−.047 (.034)	−.040 (.034)	.051 (.040)	.030 (.035)	.137 (.092)	.124 (.084)	−.078 (.052)	.024 (.050)
TORTa	.132 (.045)†	.065 (.054)	.011 (.034)	.018 (.034)	−.013 (.040)	.046 (.035)	.110 (.094)	−.144 (.081)	.049 (.051)	.005 (.050)
TORTv	−.026 (.045)	−.016 (.054)	.000 (.034)	−.030 (.034)	−.021 (.040)	.018 (.035)	.055 (.092)	.020 (.080)	.087 (.050)	−.085 (.048)
BCa	.051 (.048)	−.017 (.058)	.024 (.036)	.014 (.035)	−.034 (.042)	043 (.037)	−.126 (.100)	.146 (.092)	.042 (.054)	−.068 (.054)
BCv	−.002 (.048)	.040 (.049)	.082 (.034)†	−.095 (.035)†	−.056 (.042)	.021 (.036)	.032 (.081)	−.039 (.067)	.024 (.044)	.001 (.042)
AFa	−.060 (.048)	−.016 (.059)	.043 (.036)	−.010 (.035)	−.017 (.043)	−.058 (.037)	−.116 (.099)	.044 (.092)	.048 (.055)	−.010 (.054)
AFv	−.049 (.048)	.058 (.056)	.056 (.035)	−.021 (.035)	.053 (.042)	−**.129 (.035)**∗	.043 (.096)	−.007 (.077)	.068 (.051)	−.003 (.049)
LDRa	.080 (.048)	.019 (.058)	.015 (.036)	−.046 (.035)	.042 (.093)	.088 (.037)†	−.144 (.097)	.152 (.093)	−.018 (.018)	.017 (.054)
LDRv	.002 (.047)	.106 (.056)	.057 (.035)	−.024 (.035)	−.013 (.042)	−.028 (.036)	−.213 (.089)†	−.043 (.079)	.021 (.053)	.141 (.050)†

NOTE. Bolded values are statistically significant.

Abbreviations: g, general factor; SE, standard error; CRAE, central retinal arteriolar equivalent; CRVE, central retinal venular equivalent; BSTDa, standard deviation of arteriolar widths in zone B; BSTDv, standard deviation of venular widths in zone B; FDa, arteriolar fractal dimension; FDv, venular fractal dimension; TORTa, arteriolar tortuosity; TORTv, venular tortuosity; BCa, arteriolar branching coefficient; BCv, venular branching coefficient; AFa, arteriolar asymmetry factor; AFv, venular asymmetry factor; LDRa, arteriolar length-to-diameter ratio; LDRv, venular length-to-diameter ratio.

∗ *P* < .001; all *P* values corrected for false discovery rate.

† Value was statistically significant at *P* < .05 before FDR correction.

‡ Categorical predictor; all other predictors continuous.

## 4 Discussion

Our study provides data on a wide array of retinal vascular features, addressing the question of whether individual differences in these measurements are associated with differences in the age 73 levels of, and age 73–79 changes in, cognitive abilities. Our results provide no evidence to support the use of quantitative retinal vascular measurements to predict nonpathological cognitive aging in a relatively healthy sample of this age and background. There were very few associations between retinal predictors and both domain-general and domain-specific cognitive levels and declines. After correcting for multiple comparisons, only one modest-sized association (*β* = −.129, *P* < .001) survived in the age- and sex-adjusted model. Our results are consistent with previous null results from cross-sectional analyses of the milder retinal vascular changes analyzed in the currently used LBC1936 cohort [6], [7], [8].

The lack of associations between cognition and nonpathological retinal variables may be because of the choice of retinal measurements examined in the present study. The development of pathological changes in the retinal vasculature may be a greater determinant with greater sensitivity to changes in cognitive ability in healthy sample [2], [35]. One of the conclusions drawn from the Atherosclerosis Risk in Communities Study [3] was that pathological retinal features with the strongest association with cognitive ability were those associated with blood–retinal barrier breakdown, indicators of more severe retinal microvascular disease (microaneurysms, retinal hemorrhages), suggesting an important potential pathophysiological mechanism of cognitive impairment. The strength of association with cognitive ability increases as vascular disease progresses, with relatively weak associations for vascular caliber measurements and stronger for any retinopathy [35]. The retinal measurements examined in the present study reflect milder microvascular changes [3], [35] and appear, at best, to be weakly related to cognitive ability or change. However, given the large number of people undergoing ‘nonpathological’ age-related cognitive changes, its individual differences, and the potential attraction of retinal vascular parameters as a herald of worse cognitive decline, the present study provides valuable null results.

Another potential explanation for our findings is sample selectivity; that is, we may have missed some of the less healthy individuals because they were less likely to volunteer for the study, meaning that the individuals who were recruited tended to be healthier with potentially fewer retinal vascular changes or pathologies. It is possible that this contributed to the lack of any relations of retinal variables to cognition in our sample, although effect sizes were small (*β* range −.213 to .000), and with the exception of AFv, none of these effects were close to being statistically significant after adjustment for FDR.

The follow-up period of six years is relatively brief to detect effects on cognitive trajectories, although we stress that such a period of change measured in such a large sample is quite rare. Our sample demonstrates a limited degree of cognitive change across the follow-up period. Changes over a longer period of follow-up should be examined. Follow-up retinal imaging of participants at an older age, where more severe retinal pathologies would be common, could provide a better opportunity to relate retinal features to cognition in a slightly older sample with a greater prevalence of severe cognitive impairment, where stronger associations have been found [35]. Again, it was important to establish the results at the age range and age-change period carried out in this study, in a critical decade for acceleration of cognitive loss.

To the best of our knowledge, no previous studies have examined associations between such a range of features of the retinal microvasculature and multiple domains of cognition, each assessed with multiple well-validated tests. Furthermore, our study uses latent cognitive factors rather than individual tests' scores, which enabled us to reduce the influence of measurement error [17]. The narrow age range of the LBC1936 minimizes confounding of other variables by chronological age, which can hamper the examination of predictors of within-person cognitive aging [36]. Previous studies, without the benefit of a narrow age cohort, may have inflated the apparent associations between retinal and cognitive variables.

There are some limitations that may influence the generalizability of the results. The LBC1936 is a healthy and high-functioning sample with a restricted geographic range and ethnic background. It is not possible to extrapolate from this sample to a diseased population, patients presenting to a cognitive or stroke clinic, for example, as they may have associations between retinal changes and cognitive decline, being at a more advanced stage of disease. Because this is a healthy sample, we were not able to examine the association between retinal features and cognition in a sample with vascular dementia. It may be possible that among a subgroup of patients with vascular dementia, an association between previous small-vessel structural changes and subsequent vascular dementia could be detected. There is some debate on the accuracy and repeatability of retinal measurements of semi-automated systems [37], [38], [39]. Despite retinal measurement using a validated semi-automated program (VAMPIRE), there remains an element of subjective human input which could affect measurement reliability. Furthermore, measurements from both eyes were averaged to create a mean retinal measurement. The degree of interocular symmetry remains unclear and appears to vary by measurement [7], [40], [41], which is another source of uncertainty for retinal measurement.

Though largely null, we judge the present study's results to be of value. There is increasing interest and focus in identifying early and accessible retinal biomarkers of age-related cognitive decline, and it is important to test all stages of age-related decline in this exploration, and to test healthy and disease-based groups. The present study applied robust analytic methods and had a comprehensive range of cognitive tests and retinal measurements applied to a large, age-restricted and well-characterized sample. We conclude that, given this setting and methods, quantitative retinal parameters cannot yet be used as associates of the level of cognitive abilities at age 73 years, or to predict the degree of nonpathological cognitive decline from 73 to 79.

**Research in context**

1. Systematic review: We reviewed the literature, using publication databases, focusing on studies of retinal microvasculature features and cognitive change. We found no studies examining trajectories of different cognitive domains and their associations with a wide range of retinal vascular features.
2. Interpretation: There is increasing interest in the potential of noninvasive imaging of the retina as a method of providing information about the health of the brain, and potentially, as a marker of risk for cognitive decline. Our findings provide no evidence to support the use of quantitative retinal vascular measurements to predict nonpathological cognitive change in healthy older individuals.
3. Future directions: We propose two approaches which may provide a better opportunity to relate retinal features to cognition: (1) examine age-related cognitive decline over a longer period of follow-up, and (2) make use of retinal imaging of participants at an older age, when more severe retinal pathologies are more common.
