# Supplementary material for: Retinal microvascular features and cognitive change in the Lothian-Birth Cohort 1936
Source: Alzheimers Dement (Amst). 2019 Jul 10;11:500–9. doi: 10.1016/j.dadm.2019.04.012 (PMC6625967; doi:10.1016/j.dadm.2019.04.012)
Supplement: Supplementary Material [file mmc1.docx]

*Supplementary Table S1.* Retinal vascular parameters measured for each retinal image

| Parameter | Description | Summary |
| --- | --- | --- |
| CRAE | Central retinal arteriolar equivalent calibre (Patton, 2006) | Summary measure of vessel width-the 6 largest vessels within Zone B are measured |
| CRVE | Central retinal venular equivalent calibre (Patton, 2006) |  |
| BSTDa | Standard deviation of the arteriolar widths in Zone B* | Standard deviation of the widths of the arterioles used in the CRAE/CRVE calculation |
| BSTDv | Standard deviation of the venular widths in Zone B* |  |
| TORTa | Tortuosity arteriole (Annunziata, 2014) | Weighted average‡ of the tortuosity of all arterioles/venules in the entire image |
| TORTv | Tortuosity venule (Annunziata, 2014) |  |
| FDa | Fractal dimension of the arteriolar network (Stosic, 2006) | A measure of the degree of branching complexity of the retinal vasculature.  Generalised sandbox method was used to calculate multifractal dimensions D_0_, |
| FDv | Fractal dimension of the venular network (Stosic, 2006) |  |
| BCa | Branching coefficient arteriolar | The ratio of the sum of the cross-sectional areas of the two daughter vessels to the cross-sectional area of the parent vessel at an arteriolar/venular bifurcation. |
| BCv | Branching coefficient venular |  |
| AFa | Asymmetry factor arteriolar | The cross sectional area of the minor daughter divided by that of the major. |
| AFv | Asymmetry factor venular |  |
| LDRa | Length-diameter ratio arteriolar | Vessel length from the midpoint of one vascular bifurcation to the midpoint of the next bifurcation, expressed as a ratio to the diameter of the parent vessel at the first bifurcation. |
| LDRv | Length-diameter ratio venular |  |

*Zone B: an annulus 0.5-1 disc diameter from the optic disc margin. ‡ Weighted average estimates combined average tortuosity from different paths by calculating an average weighted according to the length of the different paths.

Supplementary Table S1 References

Patton N, Aslam T, MacGillivray T, Dhillon B, Constable I. Asymmetry of retinal arteriolar

branch widths at junctions affects ability of formulae to predict trunk arteriolar widths. *Invest*

*Ophthalmol Vis Sci.* 2006;47(4):1329-1333.

Annunziata R, Kheirkhah A, Aggarwal S, Cavalcanti BM, Hamrah P, Trucco ER. Tortuosity classification of corneal nerves images using a multiple-scale-multiple-window approach, in Proc. of the Ophthalmic Medical Image Analysis First Int. Workshop, pp. 113–120 (2014).

Stosic T, Stosic BD. Multifractal analysis of human retinal vessels. *IEEE Trans Med Imaging.*

2006;25(8):1101-1107.

*Supplementary Table S2*. Associations of each predictor, entered individually alongside age, sex and vascular risk factor variables with the cognitive level and slope (cognitive ageing from 73 to 79) of cognitive ability from mean age. General factor model and domain models were run separately.

| Covariate | General factor estimate (SE) | |  | Domain factor estimate (SE) | | | | | | | |
| --- | --- | --- | --- | --- | --- | --- | --- | --- | --- | --- | --- |
|  | *g* level | *g* slope |  | Visuospatial  level | Crystallized  level | Verbal memory  level | Speed  level | Visuospatial  slope | Crystallized  slope | Verbal memory  slope | Speed  slope |
| CRAE | -.099 (.053) | .114 (.058)^a^ |  | -.039 (.042) | -.023 (.039) | -.082 (.047) | .019 (.043) | .072 (.092) | .044 (.082) | .034 (.054) | .050 (.052) |
| CRVE | .004 (.051) | .039 (.055) |  | -.063 (.039) | .071 (.037) | -.029 (.045) | -.002 (.041) | .081 (.086) | -.110 (.079) | .035 (.052) | .026 (.049) |
| BSTDa | -.031 (.051) | -.002 (.057) |  | .037 (.040) | -.032 (.037) | .030 (.045) | -.059 (.041) | .036 (.092) | -.053 (.082) | -.039 (.054) | .017 (.051) |
| BSTDv | .006 (.052) | .049 (.056) |  | .022 (.040) | .014 (.038) | .005 (.046) | -.038 (.041) | -.143 (.085) | -.023 (.082) | .040 (.052) | .071 (.050) |
| FDa | -.023 (.053) | .139 (.058)^a^ |  | -.047 (.041) | -.018 (.039) | -.082 (.047) | .062 (.042) | .158 (.094) | .102 (.086) | -.007 (.057) | .049 (.054) |
| FDv | -.016 (.051) | .049 (.057) |  | -.052 (.040) | -.015 (.037) | .019 (.046) | .019 (.041) | .142 (.088) | .119 (.082) | -.068 (.053) | .005 (.051) |
| TORTa | .098 (.051) | .082 (.056) |  | .029 (.040) | -.005 (.037) | -.018 (.045) | .035 (.041) | .047 (.092) | -.137 (.080) | .060 (.053) | .036 (.051) |
| TORTv | -.037 (.052) | -.025 (.058) |  | .000 (.040) | -.024 (.038) | -.058 (.046) | .025 (.041) | .103 (.092) | .029 (.082) | .072 (.054) | -.090 (.051) |
| BCa | .055 (.054) | -.045 (.060) |  | .040 (.042) | .041 (.039) | -.080 (.047) | .046 (.043) | -.098 (.095) | .086 (.090) | .039 (.056) | -.068 (.056) |
| BCv | -.028 (.055) | .040 (.056) |  | .096 (.040)^a^ | -.110 (.038)^a^ | -.057 (.048) | .020 (.043) | .031 (.082) | -.040 (.073) | .025 (.050) | .002 (.047) |
| AFa | -.041 (.055) | -.033 (.061) |  | .052 (.042) | -.021 (.040) | -.041 (.049) | -.020 (.044) | -.120 (.093) | .014 (.091) | .051 (.056) | -.014 (.055) |
| AFv | .002 (.055) | .056 (.060) |  | .071 (.042) | -.042 (.039) | .048 (.049) | -.087 (.043)^a^ | .059 (.095) | -.033 (.080) | .050 (.055) | -.013 (.052 |
| LDRa | .102 (.054) | .017 (.061) |  | .025 (.042) | -.012 (.040) | .028 (.048) | .062 (.043) | -.142 (.091) | .194 (.095)^a^ | -.055 (.055) | .021 (.055) |
| LDRv | .044 (.054) | .111 (.058) |  | .082 (.041)^a^ | -.023 (.043) | -.014 (.048) | -.023 (.043) | -.233 (.085)^a^ | .142 (.051)^a^ | .033 (.055) | .142 (.051)^a^ |

*Note.* All p-values corrected for False Discovery Rate. ^a^ = value was statistically significant at p < .05 before FDR correction. *g* = general factor; SE=standard error; SE=standard error; CRAE=central retinal arteriolar equivalent; CRVE=central retinal venular equivalent; BSTDa=standard deviation of arteriolar widths in Zone B; BSTDv=standard deviation of venular widths in Zone B; FDa=arteriolar fractal dimension; FDv=venular fractal dimension; TORTa=arteriolar tortuosity; TORTv=venular tortuosity; BCa=arteriolar branching coefficient; BCv=venular branching coefficient; AFa=arteriolar asymmetry factor; AFv=venular asymmetry factor; LDRa=arteriolar length-to-diameter ratio; LDRv=venular length-to-diameter ratio

LBC1936 Wave 2 sample

*n*=866

Participants with fundus image of the left eye

*n*=814

Participants with fundus image of the right eye

*n*=814

Participants with successful retinal image analysis of left eye

*n*=680

Participants with successful retinal image analysis of right eye

*n*=683

Participants with successful retinal measurement of both eyes

*n*=603

134 rejected due to poor image quality/too few visible vessels/known pathologies/out-of-focus/overexposure

131 rejected due to poor image quality/too few visible vessels/known pathologies/out-of-focus/overexposure

Supplementary Figure S1

Flow chart of the analytic sample used to investigate the relationship between retinal measurements and cognitive ability and change (n=603). LBC1396, Lothian Birth Cohort 19
